# Mediastinal lipomatosis as a cause of low voltage complexes on electrocardiogram and widened mediastinum: A case report

**DOI:** 10.1186/1757-1626-1-171

**Published:** 2008-09-19

**Authors:** Chethan Puttarajappa, Abhijeet Dhoble

**Affiliations:** 1 Department of Internal Medicine, Michigan State University, East Lansing, Michigan, USA

## Abstract

**Background:**

Mediastinal widening is a common finding on chest radiograph, and can be caused by numerous conditions. Most of these diseases have grave prognosis if accompanied by chest pain, and require immediate attention. However, mediastinal lipomatosis is a very benign condition caused by deposition of adipose tissue in the mediastinum.

**Case presentation:**

We present a case of a morbidly obese female patient who presented to emergency department with a fall. She had mediastinal widening on chest radiograph, and borderline low voltage on electrocardiogram. On computed tomography, mediastinal lipomatosis was evident.

**Conclusion:**

Obesity is a major epidemic in United States, and can lead to deposition of fat in the chest. Mediastinal lipomatosis is very benign condition, which rarely causes grave consequences.

## Background

Mediastinal widening is a common finding on chest radiograph, and can be caused by numerous conditions. Most of these diseases have grave prognosis if accompanied by chest pain, and require immediate attention. However, mediastinal lipomatosis is a very benign condition caused by deposition of adipose tissue in the mediastinum [1,2].

## Case presentation

**Figure 1:**
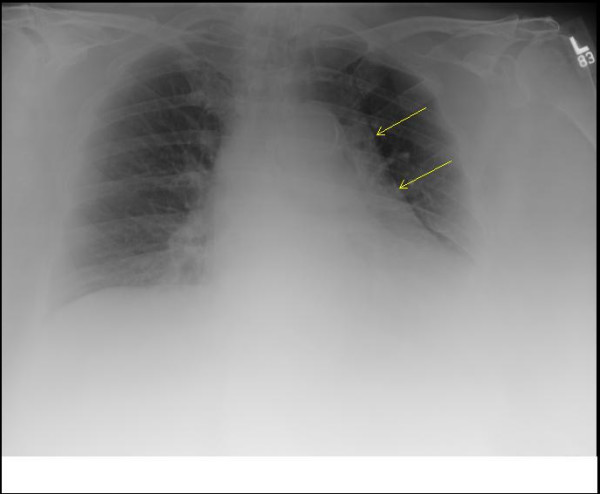
Chest radiograph showing presence of mediastinal widening with a paracardiac shadow of increased lucency giving a double contour effect.

**Figure 2:**
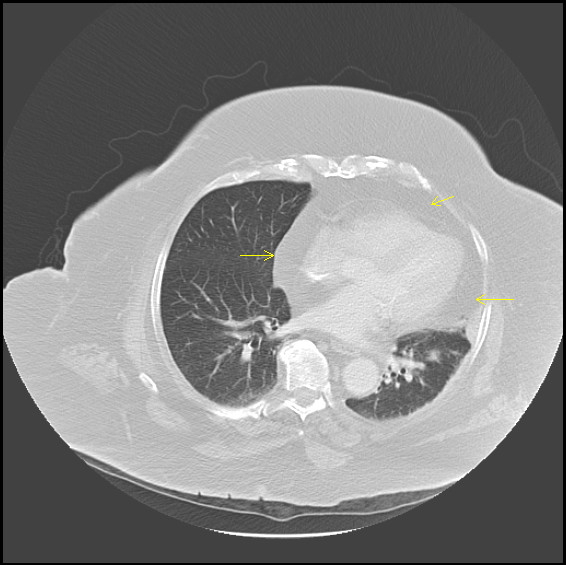
Computed tomography scan of the chest showed presence of significant fat in the mediastinum surrounding the heart and other mediastinal structures.

A 68 year old woman with morbid obesity presented to the hospital with right hip pain secondary to an accidental fall. Her body mass index was very high at 52.2. Her medical history was significant for type 2 diabetes mellitus and hypertension. She did not have any other complaints at the time of presentation. Her vital signs in the emergency department were found to be stable. An electrocardiogram was obtained on routine basis, and showed low voltage complexes. A chest radiograph (figure 1) showed the presence of mediastinal widening with a paracardiac shadow of increased lucency giving a double contour effect. A Computed tomography scan (figure 2) of the chest showed presence of significant fat in the mediastinum surrounding the heart and other mediastinal structures. No pericardial involvement was observed. A diagnosis of benign obesity associated mediastinal lipomatosis was made. The patient was completely asymptomatic and hence no therapy was initiated except counseling regarding weight loss at the time of discharge.

## Discussion

Mediastinal lipomatosis is a benign condition characterized by deposition of adipose tissue in the mediastinum. Mediastinal widening due to unusual amounts of fat accumulations is reported in the literature, which most often occurs with simple obesity, not associated with iatrogenic or primary steroid excess states [1,2]. Other causes of mediastinal widening include aortic dissection, lymphoma, thymoma, esophageal rupture, trauma or hemorrhage, and mediastinitis [3,4]. Increased amount of fat surrounding can also cause low voltages on electrocardiograms, but this has not been described in the literature.

Occasionally mediastinal fat can impose some challenges while cannulating internal jugular or subclavian catheters. There are also reports describing airway compromise due to laryngeal compression secondary to excess fat in the mediastinum [5-7].

Considering high prevalence of obesity, mediastinal widening should always be considered as a differential in evaluating patients with mediastinal widening or low voltages on electrocardiograms. Treatment usually involves weight reduction, although surgery might be needed in rare circumstances [8]. In steroid induced lipomatosis, tapering off steroids is usually needed [7]. No follow up studies are recommended.

## Conclusion

Obesity is a major epidemic in United States, and can lead to deposition of fat in the chest. Mediastinal lipomatosis is very benign condition, which rarely causes grave consequences.

## Competing interests

The authors declare that they have no competing interests.

## Authors' contributions

All authors contributed equally in collecting patient data, chart review, and editing medical images. All authors read and approved the final manuscript.

## Consent

An informed consent was obtained from the patient for publication of this case report and accompanying images in *cases journal*. A copy of the written consent is available for review by the Editor-in-Chief of this journal.
